# Ramsay Hunt Syndrome in a Patient with Rheumatoid Arthritis after Treatment with Infliximab

**DOI:** 10.1155/2014/897647

**Published:** 2014-02-09

**Authors:** Yoshio Nagayama, Naoki Matsushiro, Akihide Nampei, Hideo Hashimoto, Kenrin Shi

**Affiliations:** ^1^Department of Orthopaedic Surgery, Osaka Rosai Hospital, 1179-3 Nagasonecho, Kita-ku, Sakai 591-8025, Japan; ^2^Department of Otolaryngology, Osaka Police Hospital, 10-31 Kitayamacho, Tennoji-ku, Osaka 543-0035, Japan; ^3^Rinku Hashimoto Rheumatology Orthopaedics, 1 Rinku Orai kita, Izumisano, Osaka 598-0048, Japan; ^4^Department of Orthopaedic Surgery, Osaka University Graduate School of Medicine, 2-2 Yamadaoka, Suita, Osaka, Japan

## Abstract

A 39-year-old female patient with rheumatoid arthritis developed Ramsay Hunt syndrome after infliximab treatment. This condition is caused by the reactivation of varicella zoster virus infection in the geniculate ganglion of facial nerve in the host's immunosuppression. She was treated immediately with valaciclovir and hydrocortisone, and the complete recovery was achieved at 6 months after the onset. This is the first report of Ramsay Hunt syndrome as an adverse effect of infliximab in rheumatoid arthritis.

## 1. Introduction

Infliximab (IFX) is the firstly introduced biologic agent for the treatment of rheumatoid arthritis (RA) and its successful efficacy for the patients with methotrexate (MTX) resistant RA has been proven over ten years [1–3]. On the other hand, serious opportunistic infection and reactivation of pathogens, such as tuberculosis, *Pneumocystis jiroveci*, hepatitis B virus (HBV), cytomegalovirus (CMV), Epstein-Barr virus (EBV), and human immunodeficiency virus, have been reported to be associated with IFX treatment [4, 5].

Ramsay Hunt syndrome is characterized by herpetic vesicles in or around the ear, facial nerve paralysis, and vestibulocochlear nerve paralysis manifesting hearing loss and vertigo. It is caused by reactivation of varicella zoster virus (VZV) latently and persistently infected in the geniculate ganglion of facial nerve, often in immunosuppressed conditions of the host. Early intervention is important for complete recovery of the paralysis, but if not, it is more likely to remain permanent damage. Although several reports about VZV reactivation in RA patients have been published, reports on Ramsay Hunt syndrome in RA patients are very few. Here we report a case of Ramsay Hunt syndrome recognized in a patient with RA after treatment with IFX.

## 2. Case Report

A 39-year-old woman, diagnosed as RA in 1997, had been treated with MTX, bucillamine, and prednisolone, but unfortunately was resistant to these medications. In May 2005, her disease activity of RA elevated as high as Disease Activity Score including a 28-joint count (DAS28 [6])-erythrocyte sedimentation rate (ESR) of 5.60. Moreover, the progression of joint destruction and functional status was recognized as stage III and class III by Steinbrocker's classification [7, 8], respectively. Then IFX, a biologic agent inhibiting tumor necrosis factor (TNF)-*α*, was introduced with a dose of 3 mg/kg. After three times of IFX administration at 0, 2, and 6 weeks, DAS28-ESR improved significantly to 3.96.

**Figure 1:**
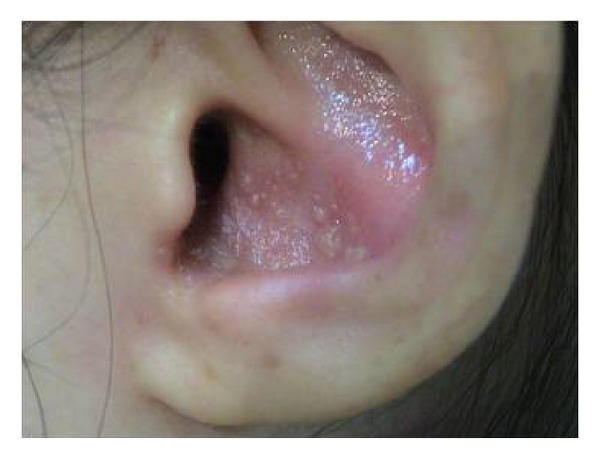
Vesicles on the left pinna.

**Figure 2:**
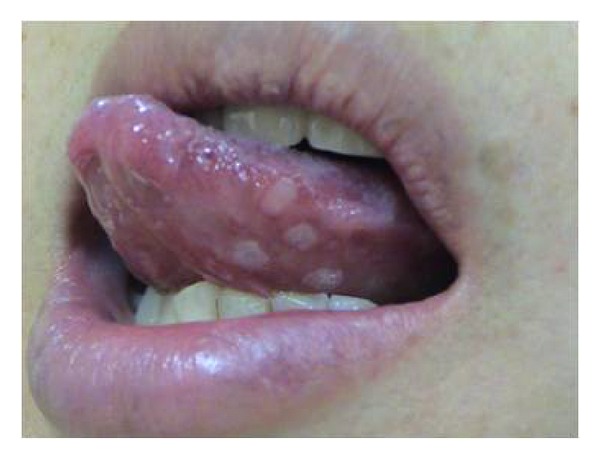
Vesicles on the left side of tongue.

Ten days after the third infusion of IFX, she felt sore throat, dysphagia, and left otalgia, with development of vesicles on the left pinna. Then three days after these initial symptoms, she felt motor paralysis on the left side of the face. She visited otolaryngologist in a private clinic who immediately consulted the otolaryngology department in our hospital at the same day; thereafter, she was diagnosed with Ramsay Hunt syndrome. Physical examination presented multiple vesicles on the left pinna, as well as on the left side of soft palate, tongue, and epiglottic vallecula (Figures 1 and 2). Facial nerve score was 4 points with Yanagihara' grading scale ([9]; total, 40 points). Neurootological examination presented sensorineural hearing loss (36.3 dB; normal value, ≤30 dB), nystagmus, and diminished lacrimation (Schirmer test, 7 mm; normal value, ≥10 mm) on the left side, with the disappearance of the left stapedial reflex and the reduced sense of taste (electrogustometry, 32 dB; normal value, ≤8 dB). Since such symptoms as sternocleidomastoid muscle atrophy, trapezius muscle paralysis, neck stiffness, and Kernig's sign were not recognized, either accessory nerve paralysis or meningitis was not suggested. Serological study by enzyme immunoassay (EIA) for specific antibodies against VZV presented IgM 0.45 (normal value, <0.80 antibody index) and IgG 29.3 (normal value, <2.0 EIA titer). At 12 days after the onset, increased levels of VZV antibodies were recognized: IgM 2.02 and IgG 83.9 (Table 1).

**Figure 3:**
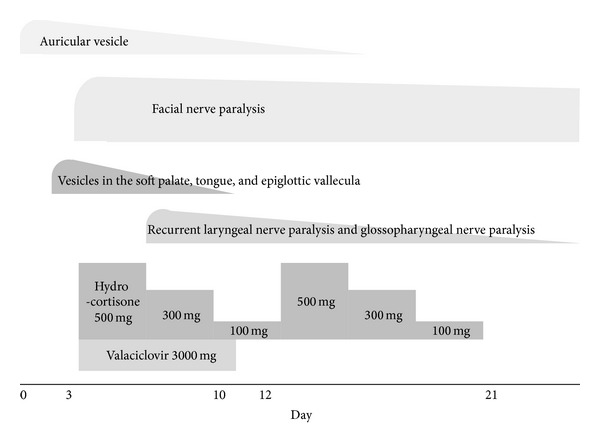
Initial clinical course of Ramsay Hunt syndrome. At 3 days after the onset, the treatment was started with valaciclovir 3000 mg for 7 days and hydrocortisone sodium succinate for 9 days (500 mg for 3 days, 300 mg for 3 days, and 100 mg for 3 days), followed by another course of hydrocortisone sodium succinate with the same dose for 9 days. Vesicles, recurrent laryngeal nerve paralysis, and glossopharyngeal nerve paralysis were relieved soon after the treatment but facial nerve paralysis continued.

**Figure 4:**
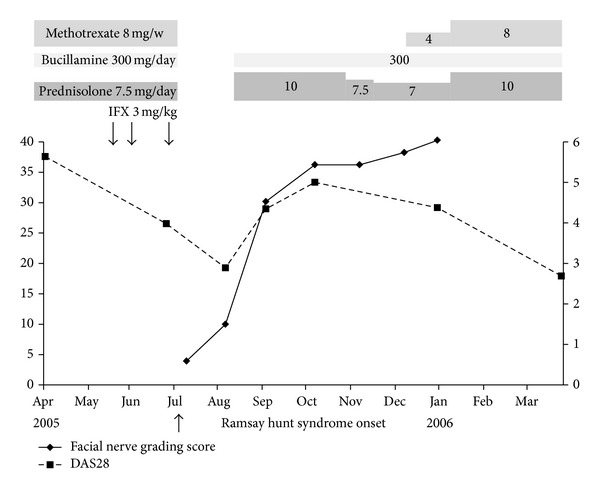
Clinical course of facial nerve paralysis and RA. Facial nerve paralysis as graded by Yanagihara's scale recovered completely at 6 months after onset, except for a slight synkinesis. DAS28-ESR elevated after discontinuation of RA treatment but relieved after readministration of medications including MTX. RA: rheumatoid arthritis; DAS28: Disease Activity Score including a 28-joint count; ESR: erythrocyte sedimentation rate; MTX: methotrexate.

Treatment was started immediately after her visit at our hospital with valaciclovir 3000 mg for 7 days and hydrocortisone sodium succinate for 9 days (500 mg for 3 days, 300 mg for 3 days, and 100 mg for 3 days), while all medications for RA, IFX, MTX, bucillamine, and prednisolone were discontinued. At 6 days after the onset, left recurrent laryngeal nerve paralysis and left glossopharyngeal nerve paralysis, the symptom of which is characterized as “curtain sign”, appeared. At 7 days after the onset, electroneurography (ENoG) and nerve excitability test (NET) were performed for prognostic diagnosis [10], and ENoG was 24.5% (excellent, ENoG ≥ 40%; good, 20% ≤ ENoG < 40%; fair, 10% ≤ ENoG < 20%; poor, ENoG < 10%), while NET was 4.2 mA (good, NET ≤ 3.5 mA; fair, 3.5 mA < NET ≤ 20 mA; poor, NET > 20 mA), both indicating unfavorable prognosis of paralysis. Then another course of hydrocortisone sodium succinate with the same dose for 9 days was conducted (Figure 3). After these treatments were introduced, facial nerve paralysis, as graded by Yanagihara's scale, improved successfully to 36 points at 3 months after the onset, and finally to full marks at 6 months (Figure 4). Other cranial nerve symptoms recovered at 2 months after the onset. However, facial synkinesis appeared at 3 months after the onset and remained even at 6 months, though it improved gradually to low grade. As for serological study, levels of VZV antibodies demonstrated a tendency to decrease after the treatment was introduced, and IgM level declined to normal value, 0.55, at 2 months after the onset whereas IgG level still remained high, 47.6 (Table 1).

With regard to treatment of RA, prednisolone 10 mg/day and bucillamine 300 mg/day were readministered at 26 days after the onset of Ramsay Hunt syndrome. Then MTX 4 mg/week was readministered at 5 months after the onset and was increased to 8 mg/week at 6 months. Disease activity of RA elevated as high as DAS28-ESR of 4.98 at 3 months after the onset but finally improved to DAS28-ESR of 2.68 after readministration of medications, especially of MTX. Recurrence of Ramsay Hunt syndrome was not recognized, and the patient transferred to another hospital for her own reasons at 9 months after the onset (Figure 4).

## 3. Discussion

It is over ten years that biological agents such as IFX have been utilized for RA, and there have been many clinical evidences as well as experiences of their efficacy for MTX resistant RA [1–3], leading to the current general concept that they are indispensable medications for RA treatment [11–14]. However, the development of bacterial, viral, and fungal infections is one of the well-known and sometimes severe adverse events of these medications with potent immunosuppressive effect [4, 5, 15, 16], and reactivation of old or resolved infections such as tuberculosis, HBV, CMV, and EBV has been reported [6, 17–21].

Among 5040 RA patients with episodes of herpes zoster in the German biotherapy registry, TNF inhibitors were reported to be administered in 3266 patients, among which IFX was reported in 591, as compared to 1774 patients without TNF inhibitors, concluding that TNF inhibitors were significantly associated with herpes zoster (hazards ratio: 2.24) [22]. Also, in a large-scale controlled study of IFX in patients with ulcerative colitis, the incidence of herpes zoster episode increased significantly in the IFX group (1.3%), as compared to the placebo group (0.4%) during 54 weeks [23].

Ramsay Hunt syndrome is a rare condition which is caused by reactivation of latent or persistent infection of VZV in the geniculate ganglion of facial nerve, occurring mostly in the host's immunosuppression [24, 25]. Its major symptoms are herpetic vesicles in or around the ear, facial nerve paralysis, and vestibulocochlear nerve paralysis presenting hearing loss and vertigo, and the annual incidence was reported previously as 5/100,000 person-year [26], much less than that of herpes zoster reported as over 300/100,000 person-year [27, 28]. Moreover, there is only one case report of Ramsay Hunt syndrome occurring in a patient with RA under etanercept treatment [29], and there have so far been no reports on Ramsay Hunt syndrome in RA patients after IFX treatment.

As the grade of facial nerve paralysis in Ramsay Hunt syndrome is severe, the prognosis is worse, and early treatment is thought necessary for complete recovery [30]. Among 28 patients in whom the treatment was started within 3 days from the onset of facial nerve paralysis, complete recovery was observed in 21 patients (75%), while it was observed in 14 (48%) among 29 patients in whom the treatment was started at 4 to 7 days after the onset. Furthermore, among 23 patients in whom the treatment was started later than 7 days, the complete recovery was only in 7 (30%) and permanent as well as none or less severe paralysis remained in other patients [31].

In the case presented in this paper, it is assumed that inhibition of TNF-*α* by IFX promoted the reactivation of latently infected VZV in the geniculate ganglion of facial nerve and caused Ramsay Hunt syndrome. Fortunately, an otolaryngology specialist appropriately diagnosed and the treatment was started as early as at 3 days after the onset, after which the complete recovery of the paralysis was achieved. In conclusion, this is the first report of Ramsay Hunt syndrome as an adverse effect following IFX treatment. Rheumatologists should be aware of this condition, especially for the patients with immunosuppressive treatments. Also, when this condition is suspected, immediate treatment should be introduced after the proper diagnosis by an otolaryngology specialist.

## Figures and Tables

**Table 1:** Serological study of varicella zoster virus (VZV).

	Day 3	Day 12	Day 25	Day 62
IgM (antibody index)	0.45	2.02	1.35	0.55
IgG (EIA titer)	29.3	83.9	68.6	47.6

Values of specific antibodies against VZV, IgM, and IgG are shown in a time-course manner. Antibodies were studied by enzyme immunoassay (EIA). Normal value; IgM: <0.80 antibody index; IgG: <2.0 EIA titer.
